# An Updated Framework for Modeling White‐Tailed Deer (*Odocoileus virginianus*) Habitat Quality in Illinois, USA

**DOI:** 10.1002/ece3.70487

**Published:** 2024-11-03

**Authors:** Jameson Mori, William Brown, Daniel Skinner, Peter Schlichting, Jan Novakofski, Nohra Mateus‐Pinilla

**Affiliations:** ^1^ Illinois Natural History Survey University of Illinois Urbana‐Champaign Champaign Illinois USA; ^2^ Division of Wildlife Resources Illinois Department of Natural Resources Springfield Illinois USA; ^3^ Department of Animal Sciences University of Illinois Urbana‐Champaign Champaign Illinois USA; ^4^ Department of Pathobiology University of Illinois Urbana‐Champaign Urbana Illinois USA; ^5^ Department of Natural Resources and Environmental Sciences University of Illinois Urbana‐Champaign Urbana Illinois USA

**Keywords:** habitat modeling, land cover, *Odocoileus virginianus*, white‐tailed deer

## Abstract

White‐tailed deer (*Odocoileus virginianus*) are a cervid species found mostly in the Americas. Managing white‐tailed deer requires understanding their relationship with the environment, which was characterized by Roseberry and Woolf (Wildlife Society Bulletin **1**, 1998, 252) for all counties in Illinois, USA, who incorporated habitat quantity and quality in a deer habitat suitability index. However, this index was based on satellite imagery from 1996 and did not explore the smaller spatial scales used by deer. Our study addressed these gaps by developing a deer land cover utility (LCU) score for each TRS (township, range, and section), township, and county in Illinois based on the methodology outlined in Roseberry and Woolf (Wildlife Society Bulletin **1**, 1998, 252) but using data from the National Land Cover Database (2001–2021). These deer LCU scores were validated against minimum deer population data using Bayesian regression with additional covariates relevant to hunting and deer density. These models performed well with Bayesian *R* ^2^ values of 0.501 (TRS), 0.5 (township), and 0.969 (county). The regression coefficients for the deer LCU scores were statistically significant (95% credibility interval not containing 0) and positive at the TRS, township, and county levels, reflecting the expected relationship between minimum deer density and deer LCU. Predictions made by these regression models on new data were accurate, with the median absolute difference between the true and predicted values being 0.398 deer/km^2^ for TRS', 0.085 deer/km^2^ for townships, and 0.066 deer/km^2^ for counties. This deer LCU could be used in other studies about deer in Illinois or studies in which deer are a relevant factor such as investigations about deer disease or tick distribution. This modeling approach could also be adapted to different wild species, locations, and/or time periods for which land cover data is available.

## Graphical abstract

A white‐tailed deer land cover utility (LCU) score was developed for each TRS (township, range, and section), township, and county in Illinois, USA. This metric was based on a quality and quantity assessment of National Land Cover Data for all datasets released to date (2001–2021). This deer LCU score can be used in future modeling efforts that require habitat information for white‐tailed deer and the methods used to calculate it can be adapted to other locations and time periods.

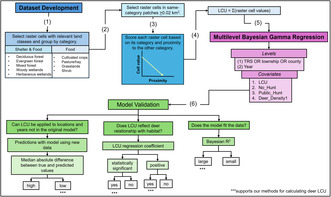

## 1 Introduction

White‐tailed deer (*Odocoileus virginianus*) are a cervid species native to North, Central, and South America (Nixon et al. 1991) that have also been introduced to Europe (Poutanen, Wikström, and Brommer 2022), New Zealand (New Zealand Department of Conservation 2024), and some Caribbean islands (Keehner, Cruz‐Martinez, and Knobel 2016). In the midwestern United States, these cervids have flourished in the fragmented landscape created by human development and row crop agriculture after populations were allowed to recover from overhunting in the mid and late 20th century (Nixon et al. 1991; Smith 1991). White‐tailed deer favor edges where different habitat types meet, particularly where forest abuts agricultural fields (Nixon et al. 1991; Smith 1991). Patches of natural areas thus provide shelter, food, and areas to reproduce, while surrounding agriculture and other open areas provide abundant supplemental nutrition almost year‐round (Nixon et al. 1991; Smith 1991). This supplemental food source and optimal landscape configuration allow Illinois deer to be exceptionally healthy (Nixon et al. 1991), as evidenced by the high pregnancy rates observed in Illinois fawns of 20.5% (Green et al. 2017).

The robust recovery of the white‐tailed deer population in the US Midwest and many other places these animals live means that humans must manage these populations to maintain healthy population levels and reduce deer‐associated problems like crop depredation (Smith 1991), over‐browsing of natural plants (Kain et al. 2011; Wilbur et al. 2017), and deer‐vehicle accidents (Hussain et al. 2007; Muller et al. 2014). Management can take many forms, but to be successful, any management method must incorporate knowledge of the environment, the deer population, and how deer utilize the land. It would therefore be useful to have a metric to characterize white‐tailed deer habitat that could be used by wildlife managers and researchers to better understand the distribution and density of these deer on the landscape.

The development of a metric that accurately identifies, quantifies, and qualifies deer habitat is necessary for all deer management and research purposes that require an understanding of and accounting for deer ecology, behavior, and distribution on the landscape. This understanding is applicable to investigations of disease cycles involving deer that are relevant to human, livestock, and domestic animal health, such as tick‐borne pathogens (Pfäffle et al. 2013; Tsao et al. 2021). An updated deer habitat metric would also assist with deer management by providing information for deer population estimates, allowing further investigation of factors influencing deer densities (Urbanek and Nielsen 2013; DeYoung et al. 2019; Hanberry 2021), contact networks (Koen et al. 2017), home range sizes (Vercauteren and Hygnstrom 1998; Walter et al. 2011), landscape utilization (Beier and McCullough 1990) and conflict with humans (Nielsen, Anderson, and Grund 2003; Wuensch 2024). This deer habitat metric could also provide information to aid in decisions about landscape and habitat protection or manipulation (Laurent et al. 2021).

Previous studies used satellite imagery to classify deer habitat (Congalton, Stenback, and Barrett 1993; McClain and Porter 2000), and others examined smaller‐scale behaviors using geographically weighted regression (Shi et al. 2006) or telemetry (Hiller, Campa, and Winterstein 2009). Studies in Mexico (Bolívar‐Cimé and Gallina 2012) and Arkansas (Miranda and Porter 2003) explicitly calculated deer habitat suitability indices based on different landscape classification methods. A study of white‐tailed deer in Illinois looked at the characteristics of winter habitat and the importance of landscape contiguity, particularly continuous patches of forest, for sustaining white‐tailed deer populations (Nixon, Hansen, and Brewer 1988).

Roseberry and Woolf published one widely used deer habitat model in 1998 using Illinois land cover data to develop a deer habitat suitability index (LCU)—a unitless metric with higher values indicating better habitat—by scoring habitat quantity and quality (Roseberry and Woolf 1998). Quality was assessed based on the distance between habitat patches and the preference of white‐tailed deer for edges (Waller and Alverson 1997), while extreme habitat fragmentation was penalized by excluding patches smaller than 0.02 km^2^ (Roseberry and Woolf 1998). The deer LCU was calculated at the county level and validated using linear regression against average hunter‐harvest densities, which served as a proxy for the minimum deer population density in each county.

The Roseberry and Woolf (1998) study is valuable for wildlife and habitat management, recreational hunting policies, disease mitigation, and increasing our general understanding of white‐tailed deer ecology. The methods and approaches of the Roseberry and Woolf (1998) study have been used to address topics like deer‐human relationships (Finder, Roseberry, and Woolf 1999; Locher et al. 2015), population density estimates (Santini et al. 2022), fawn survival (Vreeland, Diefenbach, and Wallingford 2004), and tick‐borne diseases (Huang et al. 2019). Furthermore, studies involving the Illinois white‐tailed deer herd often use the Roseberry and Woolf (1998) model when incorporating deer habitat into their analyses (Gonser, Jensen, and Wolf 2009; O'Hara Ruiz et al. 2013; Kelly et al. 2014), but issues arise when discussing the use of this model decades after it was published.

The Roseberry and Woolf (1998) model is based on 1996 data from the Illinois Land Cover Database and is limited in its ability to reflect the dynamic natural of land cover changes over time. In the Roseberry and Woolf (1998) model, the habitat quality/quantity scores are averaged for all years of land cover data, which could hide temporal trends and shorter‐term impacts of land class changes on deer population size and distribution. Applications for deer habitat data can require information at spatial scales smaller than a county, yet county is the only spatial unit provided in Roseberry and Woolf (1998). In the United States, a smaller spatial scale relevant to deer habitat, movement, and home ranges is the TRS (township, range, and section), which is a portion of land (median = 2.6 km^2^) designated by the US Public Land Survey System (PLSS) for the purpose of land ownership (ISGS 2003). The TRS is useful as it approximates home range sizes for females observed in the United States' agricultural Midwest of 0.3–1.7 km^2^ (Nixon et al. 1991; Etter et al. 2002; Walter et al. 2009) and 3–5 km^2^ for males (Walter et al. 2009). Analysis at the township level (median = 94 km^2^) allows for examination of a broader environmental context that can include both a deer's home range and factors beyond their home range that still impact them, like conditions surrounding deer are exposed to, anthropogenic disturbances, genetics, climate, and other such variables. Examination of data at the county level (median = 1567.3 km^2^) allows for an even broader exploration of factors influencing deer, including wildlife management practices and recreational hunting.

Having defined the spatial scales of interest, the objectives of our study were therefore to (Adams et al. 2020) develop an updated metric to represent deer habitat quality and quantity—a “land cover utility score”—for all TRSs townships, and counties based on modifications to the methods of Roseberry and Woolf (1998) and (Anderson et al. 2011) quantify the ability of the deer land cover utility score to identify deer habitat using a multilevel Bayesian Gamma regression against deer mortality data.

## 2 Methods

### 2.1 Spatiotemporal Scope and Analytical Tools

Our study includes land cover and deer mortality data collected between the years 2002 and 2022 in the state of Illinois, USA. Firearm hunting is allowed in all counties of Illinois except Cook, DuPage, and Lake counties due to the area's high human population density. The firearm hunting season takes place over the course of 7 days split between November and December. Archery hunting is allowed in all counties with firearm hunting, as well as some limited areas of counties restricted from firearms. The archery season lasts from October 1 to the end of January. Both male (“antlered”) and female (“antlerless”) deer are harvested, and all age classes may be hunted, with the Illinois Department of Natural Resources (IDNR) issuing different quotas each year by age and/or sex to meet management goals. Hunters purchase tags prior to harvesting deer and may only harvest the deer they are permitted to. All harvested deer must be reported to IDNR, and deer bagged in counties with chronic wasting disease (CWD) must be brought to an IDNR‐run check station (IDNR 2024). Hunting regulations apply to any hunting‐related activities in the state of Illinois regardless of where the hunter lives.

**FIGURE 1:**
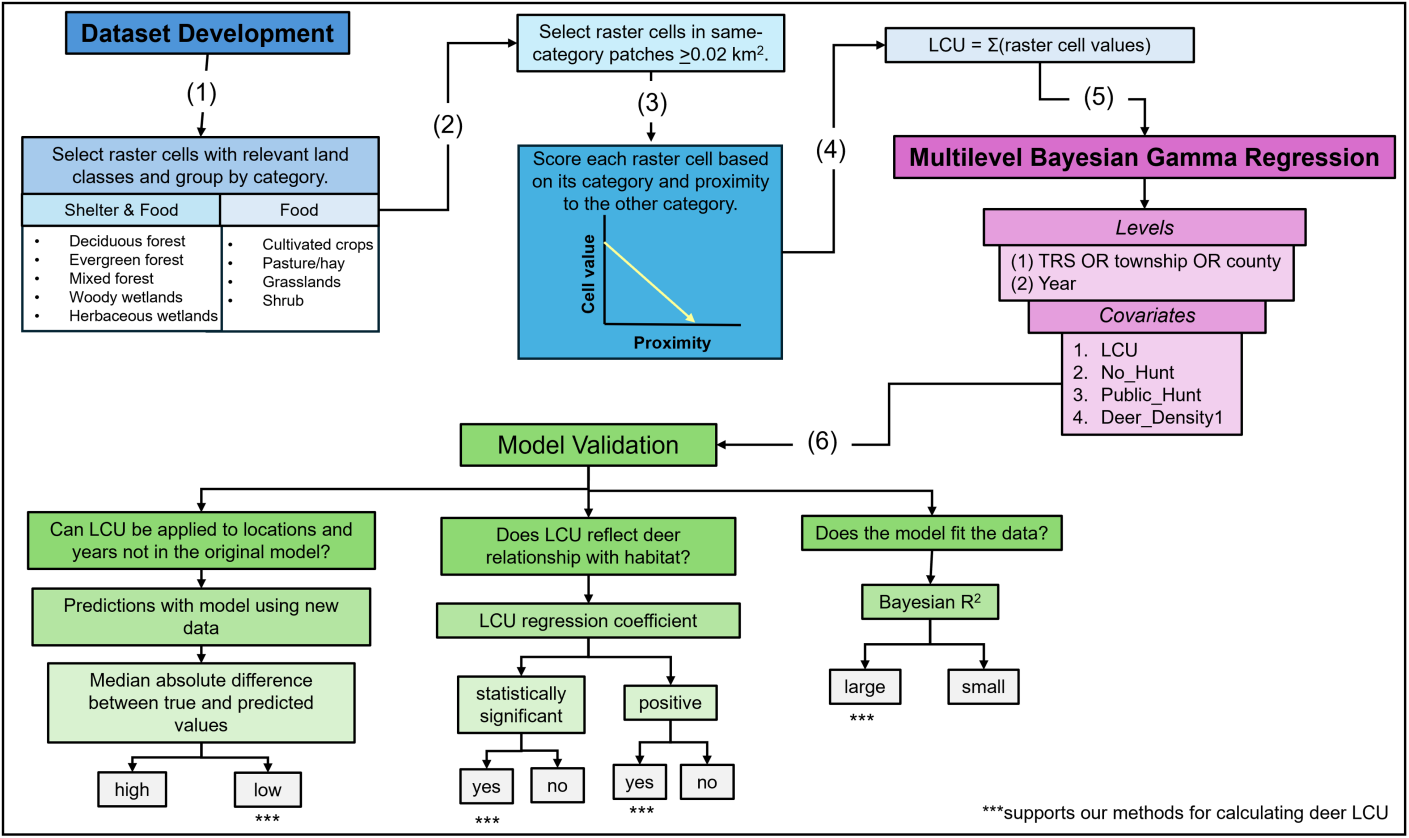
Flowchart illustrating the steps used to calculate and validate the Illinois white‐tailed deer land cover utility (LCU) score. Steps in the modeling process are grouped by colors for clarity, with dataset development in blue, multilevel Bayesian Gamma regression in pink, and model validation in green.

Spatial and statistical analyses were conducted using ArcGIS Pro (v.3.1.0) (ESRI 2023) and R (R Core Team 2023). The steps are presented in Figure 1. The datasets containing the deer LCU scores for each spatial unit are provided in the Supporting Information.

### 2.2 Approximating Minimum Deer Population Density

Deer population density estimates require information about deer presence/absence in the relevant spatial locations during the appropriate times. However, this is difficult to obtain due to the complexity of monitoring free ranging wildlife. Due to this challenge, deer population estimates are typically derived from data collected on deer mortalities. In Illinois, mortality data comes from recreational hunting, CWD‐related locally focused culling, deer population control permits, roadkill, deer suspected of illness, and deer removal permits, with hunting providing by far the most data, followed by CWD culling (Jacques and McDonal 2024). CWD‐related culling is done after the hunting season ends in areas with detected CWD with the goal of reducing the local deer population density and thereby slowing the transmission and amplification of the disease. These mortality data provide a “minimum deer population size” since at least that number of deer had to be present. This minimum deer population size is the only definite count of deer on the landscape, and so it is used to represent deer population size even though it is recognized that the true population size is larger. The minimum deer population size is then divided by the area of the spatial unit being examined to get the minimum deer density. This approach was utilized by Roseberry and Woolf (1998) and has since been validated by Adams et al. (2020).

The smallest spatial unit at which deer mortality data is available in Illinois is by TRS (township, range, and section). This data is collected by the Illinois Department of Natural Resources (IDNR) for its chronic wasting disease (CWD) surveillance program and includes deer mortalities from hunter harvest, locally focused culling (Varga et al. 2022), roadkill, and deer population control measures starting in 2002 and continuing to the present time. The majority of the CWD surveillance dataset is from areas in northern Illinois. Township‐level mortality is the aggregation of mortality values for the TRSs contained within each township.

**FIGURE 2:**
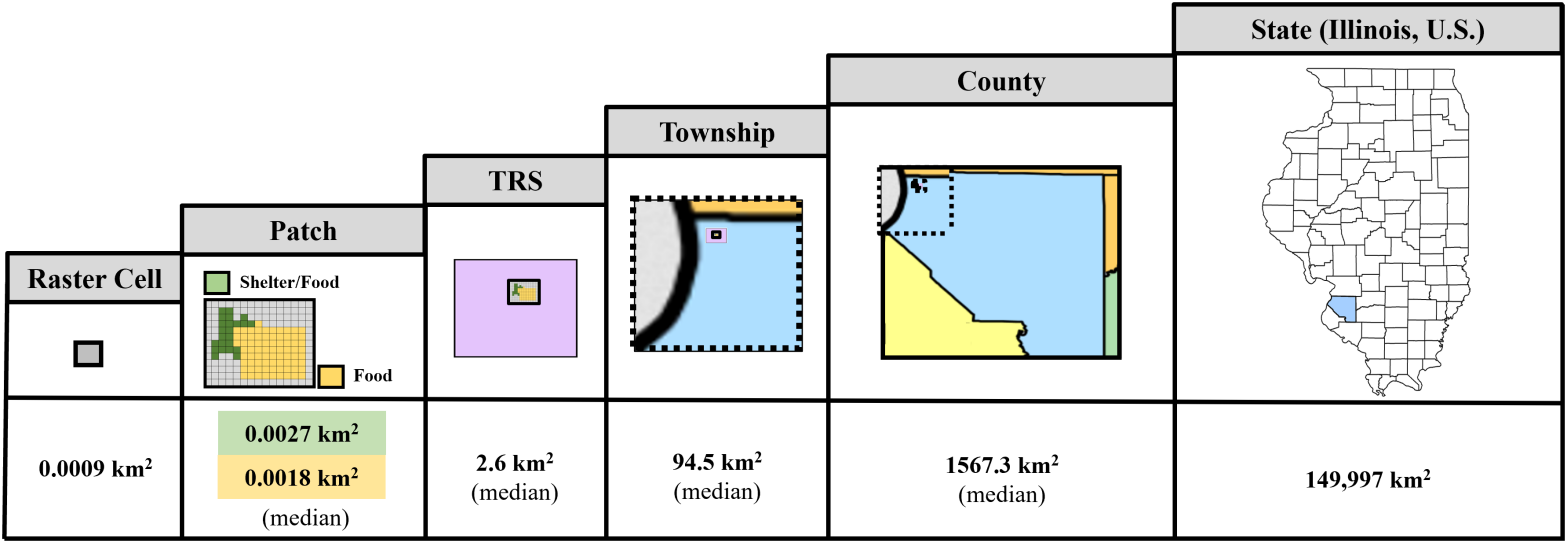
Differences between the spatial units used in this study. The size of the spatial unit is listed below the image, and the median size is reported if there are multiple values. The relative sizes of the images are not representative of the actual relative sizes of the units.

Recreational hunter harvest data collected annually by IDNR serves as the source of mortality information at the county level. This source is used for its inclusion of all counties in Illinois for all years examined, which is a stronger option than aggregating the CWD surveillance dataset by county. The hunter harvest data is available on the IDNR's White‐Tailed Deer Illinois website (Illinois Department of Natural Resources 2023a #1). Figure 2 illustrates the difference between the spatial units involved in the study.

### 2.3 Land Cover Data and Selection of Land Classes Relevant to Deer Habitat

We acquired National Land Cover Database (NLCD) data for the continental United States for all release years: 2001, 2004, 2006, 2008, 2011, 2013, 2016, 2019, and 2021 (Dewitz and U.S. Geological Survey 2021). The data consisted of raster files with cell sizes of 900 m^2^ (30 × 30 m). The extent of these datasets was then limited to the border of the state of Illinois.

We selected raster cells belonging to land classes relevant to deer habitat (Table 1) and grouped them either as land that provides both shelter and food (shelter/food) or just food (food). The shelter/food group includes land types like forests that provide both food—such as forbs or grass—and protection for rest, reproductive behaviors, and offspring. In contrast, the food group includes land types like agriculture that provide food but not shelter and are seasonal in their value for deer (Nixon et al. 1991; Roseberry and Woolf 1998). We distinguish between these two groups because deer interact differently with each of them, such as utilizing row crops for food during the day but rarely sleeping in those croplands (Nixon et al. 1991; Roseberry and Woolf 1998). We note that while the land classifications in the NLCD differ from those in the 1996 satellite imagery dataset used in the Roseberry and Woolf (1998) study, the NLCD land classes were selected and grouped to preserve the original function of these relationships presented in Roseberry and Woolf (1998).

**TABLE 1:** Relationship between National Land Cover Database (NLCD) land classes and the habitat groupings used to calculate this study's deer land cover utility (LCU) score.

National Land Cover Database Land Class	Assigned Habitat Group (Mori et al. 2024)
Deciduous forest	Shelter/Food
Evergreen forest	
Mixed forest	
Woody wetlands	
Herbaceous wetlands	
Shrub/scrub	Food
Grassland/herbaceous	
Pasture/hay	
Cultivated crops	

*Note:* The National Land Cover Database (NLCD) Land Classes were the names of each land class from NLCD years 2001–2021 identified as deer habitat and included in this study. The habitat groups established in this study were “shelter/food,” which represented land classes that provide deer with both food and protection, while “food” represented land classes that only provide deer with food.

### 2.4 Minimum Patch Size Requirement

We used the above land cover classes to isolate raster cells belonging to patches of land ≥ 0.02 km^2^ (Roseberry and Woolf 1998), with a patch characterized as a collection of contiguous raster cells of the same habitat group. Contiguity means sharing a side or corner with another raster cell. We included proximal but disconnected habitat units (within 500 m of a patch), given that deer are unimpeded by mild habitat fragmentation (Nixon et al. 1991; Smith 1991), which departs from the methods in Roseberry and Woolf (1998) that removed any raster cells not part of a qualifying patch. This threshold was based on assuming the average home range for a female white‐tailed deer in Illinois (0.99 km^2^) was a perfect circle with a radius of 500 m (Walter et al. 2009).

### 2.5 Habitat Quality Scoring

Raster cells were scored for habitat quality (Table 2) based on distance to the nearest patch of shelter/food (if a food raster cell) or food (if a shelter/food raster cell). The habitat quality scores in Table 2 serve as a means of assigning a relative rank to each spatial unit, based on the final sum of these scores, and thus the exact value of each score has no inherent meaning, as was done in Roseberry and Woolf (1998). Instead, it is the comparative size of these scores and their combination with other scores in that area that matters to produce a quantity/quality scale along which all spatial units fall.

**TABLE 2:** Raster cell deer habitat quality scores (unitless) based on distance between a raster cell belonging to one habitat group and the nearest patch of the other habitat group, adapted from Roseberry and Woolf (1998).

Proximity to ≥ 0.02 km^2^ patch of…	Distance (m)	Habitat quality score
*Shelter/Food* to Food	< 500	1
	501–700	0.8
	701+	0.6
*Food* to Shelter/Food	< 200	1
	200–275	0.8
	276–350	0.6
	351–425	0.4
	426–500	0.2
	500+	0

*Note:* “Shelter/food” is defined as land classes that provide both food and protect to deer, while “food” is defined as land classes that provide only food to deer. “Shelter/Food to Food” indicates distances from a raster cell classified as “shelter/food” to the nearest patch of food, and “Food to Shelter/Food” indicates distances from raster cells classified as “food” to the nearest patch of “shelter/food”.

The gradation of land cover utility scores by proximity to the opposite habitat group assumes that habitat raster cells get less useful or accessible to deer as the distance to other patches of habitat increases. This is particularly relevant for “food” raster cells located in expansive agricultural fields, where the edges may be heavily browsed by deer, but the cores of these fields may have no deer activity at all. The scoring schema for the “food” raster cells followed Roseberry and Woolf (1998) (Table 2). However, the “shelter/food” raster cells were classified so that any cell further than 700 m away from a patch of “food” was assigned a quality score of 0.6, resulting in the inclusion of all “shelter/food” raster cells belonging to a patch ≥ 0.02 km^2^ (Table 2). This differed from Roseberry and Woolf (1998), which eliminated forest or wetlands that were > 1000 m from cropland and other forage, functionally removing the cores of large forests from consideration as deer habitat even though deer are known to inhabit those places (Waller and Alverson 1997; Nixon et al. 1991).

### 2.6 Deer Land Cover Utility (LCU) Score Calculation

Once each individual raster cell of deer habitat was scored, the deer LCU score for each TRS (LCU_TRS), township (LCU_Town), and county (LCU_County) was calculated by matching raster cells with their overlapping spatial units, summing the scores for each spatial unit/year combination, and dividing these sums by the total area of the relevant spatial unit. The datasets containing the deer LCU scores are provided in an online repository at https://doi.org/10.13012/B2IDB‐0160590_V1.

### 2.7 Deer Habitat Suitability Index (LCU) Validation

We used Bayesian multilevel regression modeling to validate the above method for calculating the deer LCU. These regressions used minimum deer population density as the dependent variable and deer LCU as a covariate, along with additional covariates outlined in Table 3 that were included to control for other factors influencing the minimum deer population density.

**TABLE 3:** Multilevel Bayesian regression covariates included in addition to the deer land cover utility (LCU) score to account for other factors impacting minimum deer population density.

Covariate	Definition	Covariate values		
		Spatial unit	Values	Units
No_Hunt	Total area of land classified as human development or open water by the NLCD, on which hunting is prohibited	TRS	0 to 3.574	km^2^
		Township	0.506 to 212.452	
		County	33.65 to 2119.29	
Public_Hunt	Count of locations at which the state of Illinois allows the public to hunt	TRS	0 to 5	sites
		Township	1 to 7	
		County	1 to 11	
Deer_Density1	Minimum deer population density of the previous year	TRS^a^	0.202 to 56.404	deer/km^2^
		Township^a^	0.001 to 5.098	
		County^b^	0.033 to 4.583	

^a^ Illinois Department of Natural Resources' (IDNR) chronic wasting disease surveillance.

^b^ Illinois Department of Natural Resources' (IDNR) recreational hunter harvest dataset (Illinois Department of Natural Resources 2023a #1).

The “No_Hunt” covariate accounted for limitations in land access and hunting, and incorporated information about how deer interact with human development. Human development can provide a haven for deer to live because hunting is forbidden near human dwellings (Harden, Woolf, and Roseberry 2005; Storm et al. 2007), which can result in areas with high human population densities appearing to have lower minimum deer densities, when deer are present and even abundant but cannot be hunted. This same human development, though, is the result of deer habitat destruction and the establishment of roads that lead to deer‐vehicle collisions that decrease deer densities (Nixon et al. 1991; Gonser, Jensen, and Wolf 2009). The same NLCD datasets used to calculate the LCU informed the “No_Hunt” parameter. The “Public_Hunt” parameter accounted for how land access affects deer density estimates and deer population size and location. The Public_Hunt data was obtained from the Hunt Illinois Application created and hosted by IDNR (Illinois Department of Natural Resources 2023b #3). We also incorporated the time‐lagged minimum deer density (“Deer_Density1”) into the models since prior mortality events (particularly recreational hunting) can be indicative of future trends and allows us to capture information about past deer herd size and location. This minimum deer density data was the same data informing the minimum deer density dependent variable obtained from the same CWD surveillance dataset.

We split this data into training (80%) and testing (20%) groups, then centered and scaled the variables by subtracting their mean and dividing by twice their standard deviation to improve model performance and allow direct comparison (Gelman 2008). Then we identified two unnested levels for use in the model—year and spatial unit (TRS, township, or county)—that accounted for similarities between observations from the same spatial unit and/or year. Bayesian multilevel regressions were constructed in R using the “brms” package (Bürkner 2017) with levels shown in brackets (Equation 1):

(1) $$\begin{array}{cc} \mathrm{Min}\;\text{deer density}\sim \mathrm{LCU}+ & \text{land with}\;\mathrm{no}\;\text{hunting}+\\ & \#\text{public hunting sites}+\min\;\text{deer density}\;\left(\text{prior year}\right)+\\ & \left[\text{Spatial Unit}\right]+\left[\text{Year}\right] \end{array}$$

We chose to use Gamma regressions with log‐link functions since minimum deer density can take values from 0 to positive infinity. We ran the regressions once on the training data for each spatial unit and minimum deer density dataset for 10,000 iterations with chains, cores, and thinning set to 4. The models were confirmed to have converged (stabilized at a reliable value) by examination of the Rhat statistic, which should be close to 1 if converged (Gabry and Modrák 2022). We confirmed an adequate effective sample size with a minimum effective sample size metric ≥ 0.1 (Gabry and Modrák 2022). We also calculated the Bayesian *R* ^2^ of each model and the regression coefficients.

We validated the deer LCU modeling approach for each spatial unit using these regressions through examination of three aspects: (1) regression coefficient for the deer LCU covariate, (2) model Bayesian *R* ^2^, and (3) predictive ability of the model when given new data. A statistically significant and positive relationship between the deer LCU and minimum deer density indicated that the deer LCU calculation methods sufficiently reflected how an increase in minimum deer density should accompany an increase in deer LCU. This approach was used by Roseberry and Woolf (1998) with some methodological differences. The Bayesian *R* ^2^ referenced an overall indicator of model quality and ability to explain the variation in the deer density data, and models with higher *R* ^2^ values have a better fit to the data. Lastly, we quantified the accuracy of predictions made on new data as the absolute difference between the estimate and the true value. To determine if the accuracy was sufficient or not to have confidence that the approach for calculating deer LCU would perform well enough for locations and/or years not included in the construction of the model we examined the summary statistics of these absolute differences.

## 3 Results

**FIGURE 3:**
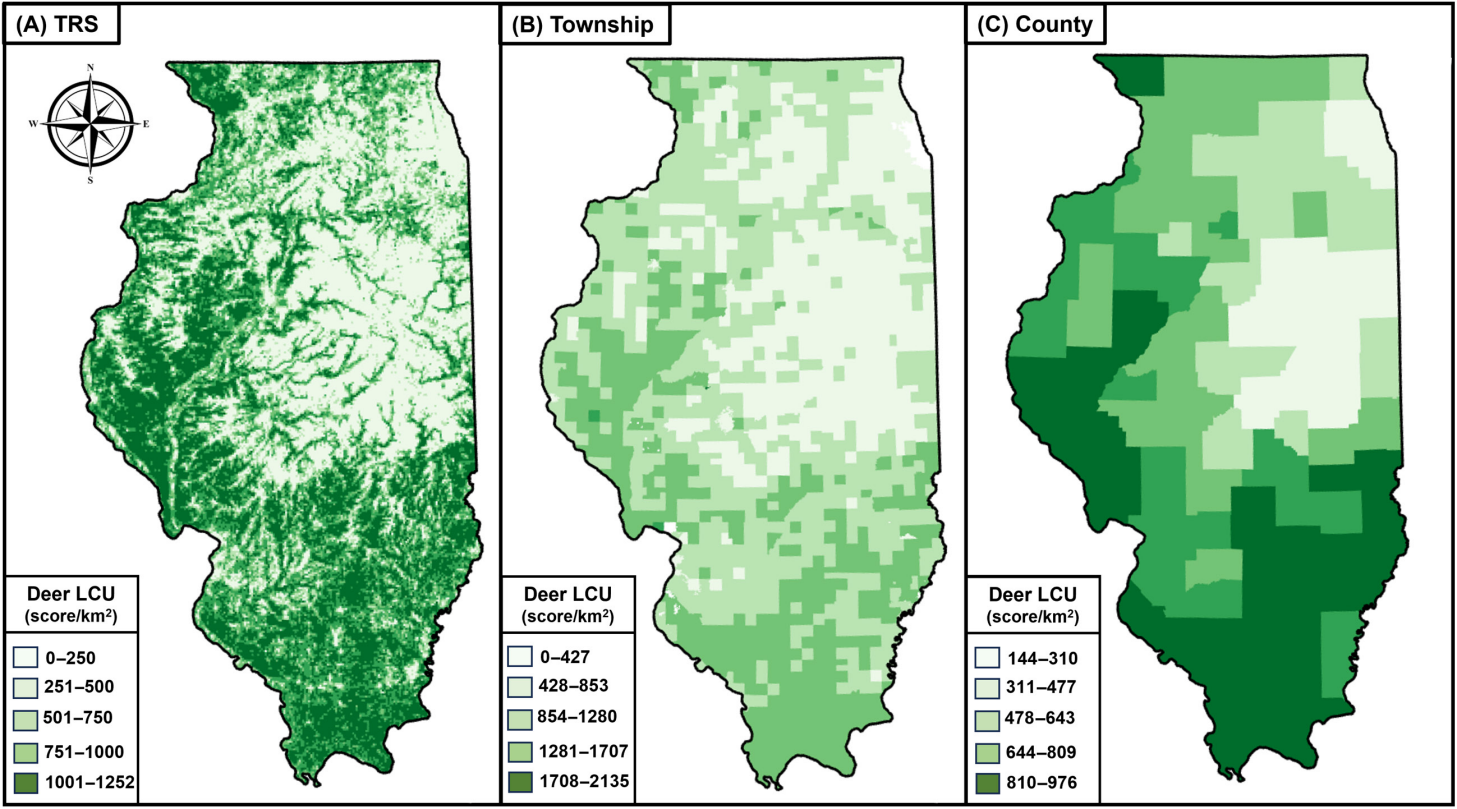
Average white‐tailed deer land cover utility (LCU) score for every (A) TRS (township, range, and section) (LCU_TRS), (B) township (LCU_Town), and (C) county (LCU_County) in Illinois, USA for the years 2001–2021.

Three datasets were generated that contained the deer LCU for each TRS (LCU_TRS), township (LCU_Town), and county (LCU_County) in Illinois, for all years included in the National Land Cover Database (2001, 2004, 2006, 2008, 2011, 2013, 2016, 2019, and 2021). The average deer LCU score is mapped by TRS (Figure 3A), township (Figure 3B), and county (Figure 3C).

The maps in Figure 3 show how finer spatial scales like the TRS can visualize habitat and environmental phenomena better than the coarser views at the broader scales of the township and county. Figure 3A in particular shows how deer habitat tends to follow water, with the outlines of rivers and streams apparent in the arrangement of LCU pixels. Trees are also important to deer habitat, with the darker shades in the south and in the northwestern corner corresponding to large expanses of forest. It is also important to observe that the general patterns of deer habitat presence and abundance hold for all three spatial levels, with concentrations in the south and along the western border.

### 3.1 Deer Land Cover Utility (LCU) Score Calculation and Validation by TRS (LCU_TRS)

In this analysis, 3820 of the TRSs in Illinois were included. The TRS‐level deer LCU (LCU_TRS) was validated through quantification of its relationship with minimum deer density using multilevel Bayesian regression that also controlled for the effects of other covariates (No_Hunt, Deer_Density1, and Public_Hunt). This regression model converged with an Rhat close to 1 and a minimum effective sample size ratio of 0.615 (> 0.1 minimum threshold). The Bayesian *R* ^2^ of this regression was 0.501, indicating that 50% of the variation in the minimum deer density data was explained by the model levels (TRS and year) and covariates. Since the model data were centered and scaled, the regression coefficients in Table 4 represent the change in minimum deer density when the covariate increases from a low to a high value. A covariate with a 95% credibility interval that does not include 0 is statistically significant. This regression had an intercept of −0.084. The regression coefficient for the TRS‐level deer LCU (LCU_TRS) model was significant and positive (0.344), supporting the use of our methods for calculating the deer land cover utility score for TRSs.

**TABLE 4:** Regression coefficient values for TRS‐level Bayesian Gamma multilevel regression (LCU_TRS).

Covariate	Rank	Regression coefficient	95% Credibility interval		Change in min deer density	
			Lower bound	Upper bound	Direction	Change (%)
Deer_Dens1	1	0.724	0.684	0.765	Increase	1.063
LCU_TRS	2	0.344	0.306	0.382	Increase	0.411
No_Hunt	3	0.21	0.17	0.249	Increase	0.234
Pub_Hunt	4	0.075	0.032	0.116	Increase	0.078

We examined the LCU_TRS model's ability to be applied to TRSs and/or years not included in the original analysis by making predictions with this model based on testing data not used to build it. Our performance metric was the absolute difference between the actual and estimated deer densities, which quantified how far from the “truth” the predictions were, with larger differences corresponding to higher error/lower accuracy. For reference, the deer densities used in our analysis ranged from 0.202 to 56.404 deer/km^2^.

The TRS‐level model yielded a minimum and maximum absolute difference of 0.0003 deer/km^2^ and 483.421 deer/km^2^, respectively. 50% of the predictions were within 0.398 deer/km^2^ of the true value and at least 75% had an absolute difference below 0.71 deer/km^2^. The mean absolute difference (1.043) was larger than both the median (0.398) and the 75th percentile (0.708). Further examination of the data set revealed that the cause of this maximum value was a single prediction, and that it was one of only six predictions with an error > 20 deer/km^2^. This suggests that there are a few data points in the testing data that were not well represented by the model developed on the training data, which is always a hazard when splitting a dataset. However, since the purpose of this analysis was to determine the overall performance of the regression model, these extreme instances can be ignored. The overall small amount of error observed in the predictions supported the application of LCU_TRS to TRSs and/or years not included in the original analysis.

### 3.2 Deer Land Cover Utility (LCU) Score Calculation and Validation by Township (LCU_Town)

In this analysis, 423 townships in Illinois were included. The township‐level multilevel Bayesian regression model (LCU_Town) converged with an Rhat around 1 and a minimum effective sample size ratio of 0.472. The Bayesian *R* ^2^ was 0.5. The model had an intercept of −1.772 and the LCU_Town covariate had a positive and statistically significant regression coefficient (0.609) (Table 5).

**TABLE 5:** Regression coefficient values for township‐level Bayesian Gamma multilevel regression (LCU_Town).

Covariate	Rank	Regression coefficient	95% Credibility interval		Change in min deer density	
			Lower bound	Upper bound	Direction	Change (%)
Deer_Dens1	1	0.949	0.801	1.096	Increase	1.583
LCU_Town	2	0.609	0.449	0.776	Increase	0.839
No_Hunt	3	0.357	0.154	0.56	Increase	0.429
Pub_Hunt	4	0.192	0.007	0.388	Increase	0.212

The absolute difference between actual and predicted values ranged from 2E−4 to 25.57 deer/km^2^, with a median of 0.085 and a mean of 0.225. This mean was larger than the median and the 75th percentile (0.189), indicating a strong skew in the data. The observation that the maximum (25.57) was much larger than the 95th percentile (0.524) suggested that only a few extreme predictions were biasing the mean and additional analysis of the data showed that this 25.57 deer/km^2^ maximum was a single, extreme value. In fact, it was the only prediction with an error above 5 deer/km^2^, and only one of four predictions with an error ≥ 1.5 deer/km^2^. As discussed in the prior TRS section, these few predictions with large errors are likely due to the model not well reflecting data points omitted from its development but these can be ignored as they have minimal influence on the aims of the study. Comparison of the median (0.085 deer/km^2^) to the range of deer densities reported in Table 3 for townships—0.0001 to 5.098 deer/km^2^—supports the conclusion that this error is small and that the LCU_Town model can be used on townships and years not included in the original construction of the model.

### 3.3 Deer Land Cover Utility (LCU) Score Calculation and Validation by County (LCU_County)

All 102 counties in Illinois were included in the multilevel Bayesian regression model that converged with an Rhat around 1 and a sufficient minimum effective sample size ratio of 0.144. This model had a Bayesian *R* ^2^ of 0.969, an intercept of −0.011, and a significantly positive regression coefficient value of 0.169 for LCU_County (Table 6).

**TABLE 6:** Regression coefficient values for the county‐level multilevel Bayesian regression (LCU_County).

Covariate	Rank^a^	Regression coefficient	95% Credibility interval		Change in min deer density	
			Lower bound	Upper bound	Direction	Change (%)
Deer_Dens1	1	0.715	0.636	0.792	Increase	1.044
LCU_County	2	0.169	0.118	0.22	Increase	0.184
No_Hunt	3	−0.599	−0.754	−0.444	Decrease	0.820
Pub_Hunt	—	0.018	−0.159	0.195	—	—

^a^ Only statistically significant covariates were assigned a rank.

The county‐level model was validated using new data and determined that the median absolute difference between true and predicted values was very low at 0.066 deer/km^2^, compared to the minimum county‐level deer density of 0.033 deer/km^2^ and maximum of 4.583 deer/km^2^ (Table 3). The range of errors was 9.03E−5 to 0.687 deer/km^2^, with a mean of 0.102, 75th percentile of 0.132, and 95th percentile of 0.269.

## 4 Discussion

This study adapted the methodology developed by Roseberry and Woolf (1998) to calculate a deer habitat suitability index (LCU) from all National Land Cover Database (NLCD) datasets released to date (2001–2021) and for all TRS' (township, range, and section) (LCU_TRS), townships (LCU_Town), and counties (LCU_County) in Illinois, USA. The methods were validated at the TRS, township, and county levels using multilevel Bayesian regression with additional covariates relevant to the estimation of minimum deer population density. The models at all three spatial scales had *R* ^2^ values equal to (TRS and township) or higher than 0.5 (county; *R* ^2^ = 0.969). These *R* ^2^ values were large enough to indicate that the regression models captured at least 50% of the variation in the minimum deer densities derived from recreational hunter harvest. This warrants confidence in the models and their reported relationships between the covariates—including deer LCU—and the minimum deer density. It should be noted that the very high *R* ^2^ for the county‐level model was due to the model structure, particularly the inclusion of the “County” and “Year” levels.

The regression coefficients for the three regression models provided key information about the ability of this updated method of calculating deer LCU to more accurately reflect minimum deer density. It was expected that a deer LCU calculated using the updated methods would have a statistically significant regression coefficient that was positive, since the minimum deer density should increase with deer habitat suitability (Roseberry and Woolf 1998). This was true for the deer LCU at all three spatial levels (LCU_TRS, LCU_Town, and LCU_County), supporting the use of these methods for updating the deer LCU calculation. We note that for all three spatial levels, the covariates had the same order of importance (min deer density (prior year) > LCU > land without hunting > # public hunting sites), and the prior year's minimum deer density and deer LCU were always positive and significant. This meant that as the prior year's minimum deer density and deer LCU increase, so does minimum deer density, which establishes that prior deer density strongly impacts future minimum deer density, and that more habitat of better quality supports higher deer densities.

Interestingly, the amount of land that cannot be hunted was always significant but was positive at the TRS and township levels but negative at the county level. A negative relationship had been expected since less space available for recreational hunting could be a constraint on the number of hunters able to access an area and the number of deer they could harvest. However, the positive coefficient at the TRS and township levels suggests other factors may be at play with the no hunting covariate. It is possible that human development provides protection from recreational hunter harvest and other deer removal methods allow deer to increase (Harden, Woolf, and Roseberry 2005; Storm et al. 2007), and that this effect is more noticeable at smaller spatial scales, which highlights a potential area of further evaluation.

The total number of public hunting sites was significant and positive at the TRS and township levels, but not at the county level, indicating that is another covariate whose effect on minimum deer density is impacted by the spatial scale at which it is considered. At the TRS and township levels, we found that more public hunting locations result in higher deer densities, likely because these locations tend to include prime deer habitat such as state parks or other prime habitats where more hunting opportunities result in more deer harvested, informing the minimum deer density dataset. The lack of significance of public hunting sites at the county level indicates that this covariate decreases in importance as the spatial scale increases and is possibly overshadowed by other covariates. The last part of the deer LCU validation was the use of the models to make predictions based on testing data to determine accuracy. The low median predictive errors for all spatial units indicated that the updated deer LCU methodology could be used for future analyses with NLCD data (or other landcover data in places like Europe) at different spatial locations and years. Overall, high Bayesian *R* ^2^ values, statistically significant and positive regression coefficients for deer LCU, and overall low error in predicting new deer densities demonstrated that this updated approach to calculating deer LCU generated a metric that properly reflected deer habitat use.

This approach to quantifying deer LCU made advances on several fronts. Part of the update to the methods of Roseberry and Woolf (1998) was the use of NLCD data, which encompasses the entire contiguous United States and is an active dataset updated every few years. Adopting a different land cover dataset that is active and updated makes it more relevant to current questions regarding deer habitat extending the value of the deer LCU methodology to other spatial locations and future times. Another advancement of the deer LCU methodology is the application to the TRS, township, and county levels, whereas previous work in Illinois was focused only on the county level (Roseberry and Woolf 1998).

This approach to quantifying deer habitat also kept years separate instead of averaging land cover values as in Roseberry and Woolf (1998) by adopting multilevel modeling with “Year” as a level. Keeping years separate did two things: it allowed for (1) the deer LCU dataset to be updated with future releases of NLCD data and (2) explicit quantification of the variation within and between spatial scales and years, which gets lost with averaging. Using the multilevel modeling approach also accounts for spatial scale and/or year‐related factors not explicitly included as covariates in the regression model, thereby improving model fit to the data and isolating the specific effects of deer LCU on deer density. Reframing land class groupings as shelter/food or food rather and employing more inclusive rules for shelter/food raster cells also recognized that deer do use core spaces in continuous forest and wetlands, even though they prefer edges (Waller and Alverson 1997). Our updates to calculating deer LCU and the LCU datasets generated expand our knowledge of deer LCU spatially—to include TRSs, townships, and counties—and temporally through use of more years of land use data. This allows a more specific look at questions of interest on a local scale and accounts for variation within and between years and/or spatial units.

This study and the deer LCU calculation methods presented here have some limitations. First, the validation of these methods was based on regression against the minimum deer density in a spatial unit and there may be alternative approaches. Second, the minimum deer density was quantified differently for the TRS and township models than it was for the county model due to their data sources, with the TRS and township models restricted to deer mortalities reported in Illinois' CWD surveillance dataset, which is not exhaustive of all the TRSs and townships in Illinois. It would be good to repeat this analysis should a more comprehensive data set become available. However, enough TRSs and townships were represented to allow a sufficient sample size for modeling, and the strong predictive ability of both models supports the use of this method on TRSs or townships not part of a CWD surveillance dataset.

In the future, the deer LCU methods should be tested at different locations in the continental United States to determine performance and broadscale application to other regions. It would also be beneficial to explore additional alterations to the deer LCU methods, such as adding other land classes or using other raster cell inclusion distances as new information arises. This would be particularly valuable in areas outside Illinois. Lastly, applying these deer LCU efforts to deer‐related models as a factor explaining deer location, behavior, density, movement, contact networks, crop predation, disease intensity or distribution, and/or deer‐vehicle collisions, or as an influence over human disease vector ecology, would be an important next step in increasing the practical importance of this metric.

## Author Contributions

**Jameson Mori:** conceptualization (equal), formal analysis (lead), investigation (lead), methodology (lead), software (lead), validation (lead), visualization (lead), writing – original draft (lead), writing – review and editing (equal). **William Brown:** conceptualization (equal), supervision (equal), writing – review and editing (equal). **Daniel Skinner:** resources (equal), supervision (equal), writing – review and editing (equal). **Peter Schlichting:** resources (equal), supervision (equal), writing – review and editing (equal). **Jan Novakofski:** funding acquisition (equal), project administration (equal), resources (equal), supervision (equal). **Nohra Mateus‐Pinilla:** conceptualization (equal), data curation (equal), funding acquisition (lead), project administration (lead), resources (equal), supervision (lead), writing – review and editing (equal).

## Conflicts of Interest

The authors declare no conflicts of interest.

## Supporting information


Data S1

## Data Availability

The deer land cover utility (LCU) scores for all TRSs, townships, and counties in Illinois, USA are provided in a data repository at https://doi.org/10.13012/B2IDB‐0160590_V1. A dataset to replicate the multilevel Bayesian Gamma regression for the county‐level deer LCU score validation process is also provided at the same link. Datasets to replicate the regressions for the TRS and township‐level analyses were not provided due to the landowner privacy concerns associated with the Illinois Department of Natural Resources' ongoing chronic wasting disease (CWD) management program, the data from which was used to inform the deer mortality variables in the regressions.
